# Depression and Anxiety after Acute Myocardial Infarction Treated by Primary PCI

**DOI:** 10.1371/journal.pone.0152367

**Published:** 2016-04-13

**Authors:** Petr Kala, Nela Hudakova, Michal Jurajda, Tomas Kasparek, Libor Ustohal, Jiri Parenica, Marek Sebo, Maria Holicka, Jan Kanovsky

**Affiliations:** 1 Department of Internal Medicine and Cardiology, University Hospital Brno, Brno, Czech Republic; 2 Faculty of Medicine, Masaryk University, Brno, Czech Republic; 3 Department of Psychiatry, University Hospital Brno, Brno, Czech Republic; 4 Centre of Cardiovascular and Transplantation Surgery, Brno, Czech Republic; 5 Department of Pathological Physiology, Faculty of Medicine, Masaryk University, Brno, Czech Republic; University of Bologna, ITALY

## Abstract

**Aims:**

The main objective of the study was to find out prevalence of depression and anxiety symptoms in the population of patients with AMI with ST-segment elevation (STEMI), treated with primary PCI (pPCI). Secondary target indicators included the incidence of sleep disorders and loss of interest in sex.

**Methods and results:**

The project enrolled 79 consecutive patients with the first AMI, aged <80 years (median 61 years, 21.5% of women) with a follow-up period of 12 months. Symptoms of depression or anxiety were measured using the Beck Depression Inventory II tests (BDI-II, cut-off value ≥14) and Self-Rating Anxiety Scale (SAS, cut-off ≥ 45) within 24 hours of pPCI, before the discharge, and in 3, 6 and 12 months). Results with the value p<0.05 were considered as statistically significant. The BDI-II positivity was highest within 24 hours after pPCI (21.5%) with a significant decline prior to the discharge (9.2%), but with a gradual increase in 3, 6 and 12 months (10.4%; 15.4%; 13.8% respectively). The incidence of anxiety showed a relatively similar trend: 8.9% after pPCI, and 4.5%, 10.8% and 6.2% in further follow-up.

**Conclusions:**

Patients with STEMI treated by primary PCI have relatively low overall prevalence of symptoms of depression and anxiety. A significant decrease in mental stress was observed before discharge from the hospital, but in a period of one year after pPCI, prevalence of both symptoms was gradually increasing, which should be given medical attention.

## Introduction

Depression or anxiety and ischemic heart disease (IHD) significantly more often occur together.[1–4] In the meta-analytical study of Thombs et al.[1], a structured interview identified depressive disorder on average in 20% of patients after acute myocardial infarction (AMI); depression symptoms identified by the BDI questionnaire (Beck Depression Inventory) (score ≥ 10) were present in approximately 31% of patients. In the general population, depressive disorder (according to the DSM-III-R criteria) is found in 4.5%–9.3% of women and 2.3%–3.2% of men[5], and depression symptoms, but also depressive disorder are considered normal conditions after myocardial infarction (MI)[5]. In the first week after percutaneous coronary intervention (PCI), the prevalence of anxiety ranges around 25–37% [6], but up to 67% of patients after PCI may be depressed[7]. A significant portion of the data in the studies, however, is based on the evaluation of self-report questionnaires, and is often influenced by topical non-specific feelings (“unease”).

The first symptoms of depression appear between 48 and 72 hours after MI and in most patients disappear within 5 or 6 days[4]. Damen et al.[8], describing intra-individual changes in depression and anxiety during the one-year follow up of patients after PCI, found that both variables are stable over time. 81% of patients after 12 months still had symptoms of depression, and 76% of patients still had symptoms of anxiety. The higher the measured score of both variables at the beginning, the more likely this score remained increased even after 12 months.

The study of the incidence of depression and anxiety symptoms is important because of the potential impact of these variables on subsequent morbidity and mortality of patients. In 1993, Frasure-Smith et al.[9] as the first ones identified depression after MI as an important predictor of 3-4-fold increase in mortality from cardiac causes, regardless of previous MI’s and/or left ventricular dysfunction. Opinions concerning the influence of depression on higher mortality at the time of recovery after AMI vary; some of them confirm this relationship[10–12], others do not[13,14]. A recent study confirmed that depression 1.6-times increased the likelihood of death in the long term horizon of 7 years after PCI[15]. However, it is not certain what causal relationship this is, as treatment with antidepressants (sertralin) or with cognitive behavioural therapy (CBT) has never been associated with a favourable effect[16,17]. Possible explanations of more frequent co-occurrence of depression and IHD are being sought in more frequent occurrence of unhealthy lifestyles in depressive people[18], non-adherence to treatment[19], later seeking medical care[20], increased activation of the hypothalamic-pituitary-adrenal (HPA) axis[21], increased activation of platelets[22], abnormal endothelial function[23], etc.

The main objective of this study was to determine the prevalence of depression and anxiety symptoms in the population of patients with AMI with ST-segment elevation (STEMI) treated by currently the most effective method, which is a mechanical reperfusion using primary PCI (pPCI). This study is unique by the number of measurements throughout the year, which allows us to track trends over time. Secondary objectives were to determine co-morbidity of depression and anxiety and the difference between men and women in the level of anxiety and depression experienced during the recovery.

## Materials and Methods

The study protocol was approved by the Ethical Committee of the University Hospital Brno and conforms to the ethical guidelines of the 1975 Declaration of Helsinki.

Prospectively, 79 consecutive patients were enrolled in the study over 16 months, with STEMI treated by pPCI within 12 hours of the onset of problems and after signing informed consent. Exclusion criteria included AMI in the medical history, the inability to complete the questionnaire, the impossibility of finishing the 12-months follow-up, a severe chronic disease with poor prognosis (e.g. malignancy, more severe cerebral stroke, organ complications of diabetes, etc.), and the age over 80 years. These patients were followed up in the period of 1 year after AMI in five different time intervals (within 24 hours from pPCI, before discharge from the hospital, in 3, 6, and 12 months). The project was approved by the local Ethics committee.

The symptoms of depression were measured by means of BDI-II inventory. The Czech BDI-II version was validated by Preiss and Vacíř[24]. This self-rating scale with 21 items, which does not take much time to complete (5–10 minutes), is focused on depression symptoms: affective, motivational, cognitive, and physiological. For each of 21 items, the respondent ticks the statement that best describes how he/she felt during the last 14 days. Except for two items (16 and 18), the items are on a four-point scale. Items 16 and 18 have a seven-point scale. The minimum score is 0, and the maximum 63. From 14 to 19 points (14 and 19 including), the score is interpreted as slightly depressive, 20 to 28 points as moderately depressive, and 29 to 63 as severely depressive symptoms[24].

Symptoms of anxiety were measured using the inventory of SAS (Zung’s Self-Rating Anxiety Scale) [25]. It is again a method that does not take much time; it contains 20 items, identifying affective and physiological symptoms of anxiety. The final score describes, similar to BDI-II, the condition in the last two weeks, not anxiousness or a depressive state of mind as a personality trait. The respondent uses a four-point scale to assess all statements (from never / rarely, to very often / all the time). The score can be from 20 to 80 points. As a cut-off score, it is recommended to use the level of 45 points [25].

We subjected two areas, which are assessed by the self-rating scales, to a special analysis, namely sleep disturbances and loss of interest in sex.

The data was analyzed in the Statistika Cz program, version 12, and in MS Office—Excel 2007. Results with the value p<0.05 were considered as statistically significant. Mann-Whitney U tests were used to compare those who withdrew from the study during its course and those who continued. When analyzing BDI-II and SAS as continuous variables, the Spearman correlation coefficient was calculated to determine the relationship between the initial and subsequent rate of depression and anxiety. The same method was used to compare co-morbidity or depression and anxiety symptoms. To determine the difference between the values of both tests for men and for women, the Mann-Whitney U test was used. Kruskal Wallis ANOVA was used to determine the relationship between age groups and values of BDI-II and SAS.

## Results

The research involved 79 patients (78.5% of men; median age 61 (5^th^ percentile 43.6, 95^th^ percentile 77.0, range [32–79] years).

### Occurrence of depression symptoms

Within 24 hours after pPCI, depression symptoms (BDI-II ≥ 14) were identified in 17 patients (21.5%). In the time before the discharge (in 3–5 day after pPCI), questionnaires were handed in by 76 patients (96.2%), 7 patients (9.2%) were with depression symptoms. After 3 months, 67 patients (84.8%) responded. Of the remaining patients, 7 patients (10.4%) were with depression symptoms. After 6 months, 65 patients (82.3%) responded, 10 of this patients (15.4%) were with depression symptoms. And during the last measurement after 12 months, 9 patients of 65 (13.8%) with depression symptoms were found. (Table 1, Graph 1) There were no differences between patients who did or did not follow up, at any time point, (p = 0.51 at discharge, p = 0.27 at 3 months, p = 0.54 at 6 months), comparing to the previous questionnaire scoring.

**Table 1:** Occurrence of positivity of questionnaire BDI-II.

	*Within 24h after IM*	*Discharge*	*3M*	*6M*	*12M*
**BDI-II score ≥14 (no. of patients)**	21.5% (17/79)	9.2% (7/76)	10.4% (7/67)	15.4% (10/65)	13.8% (9/65)
**Score median (5**^**th**^**; 95**^**th**^ **percentile)**	8 (1.9; 21.0)	6 (0.0; 17.0)	5 (0.0; 15.0)	6 (0.0; 21.0)	5 (0.0; 26.4)

### Occurrence of anxiety symptoms

Within 24 hours after pPCI, anxiety symptoms (SAS ≥ 45) were felt by 7 patients (8.9%). At the time of the next measurement before the discharge from the hospital, no patient was presenting anxiety symptoms. After 3 months, 3 patients (4.5%) with anxiety symptoms were found in the group. After 6 months, 7 patients (10.8%) were with anxiety symptoms. And during the last measurement after 12 months, there were 4 patients with anxiety symptoms (6.2%). (Table 2, Graph 1). There were no differences between patients who did or did not follow up, at any time point, (p = 0.28 at discharge, p = 0.86 at 3 months, p = 0.58 at 6 months), comparing to the previous questionnaire scoring.

**Table 2:** Occurrence of positivity of questionnaire SAS.

	*Within 24h after IM*	*Discharge*	*3M*	*6M*	*12M*
**SAS score ≥ 45**	8.9% (7/79)	0% (0/76)	4.5% (3/67)	10.8% (7/65)	6.2% (4/65)
**Score median (5**^**th**^**; 95**^**th**^ **percentile)**	34 (25.0; 48.1)	34 (22.8; 42.0)	34 (21.3; 43.7)	33 (20.0; 50.4)	33 (20.2; 45.6)

### Simultaneous occurrence of depression and anxiety symptoms

**Fig 1:**
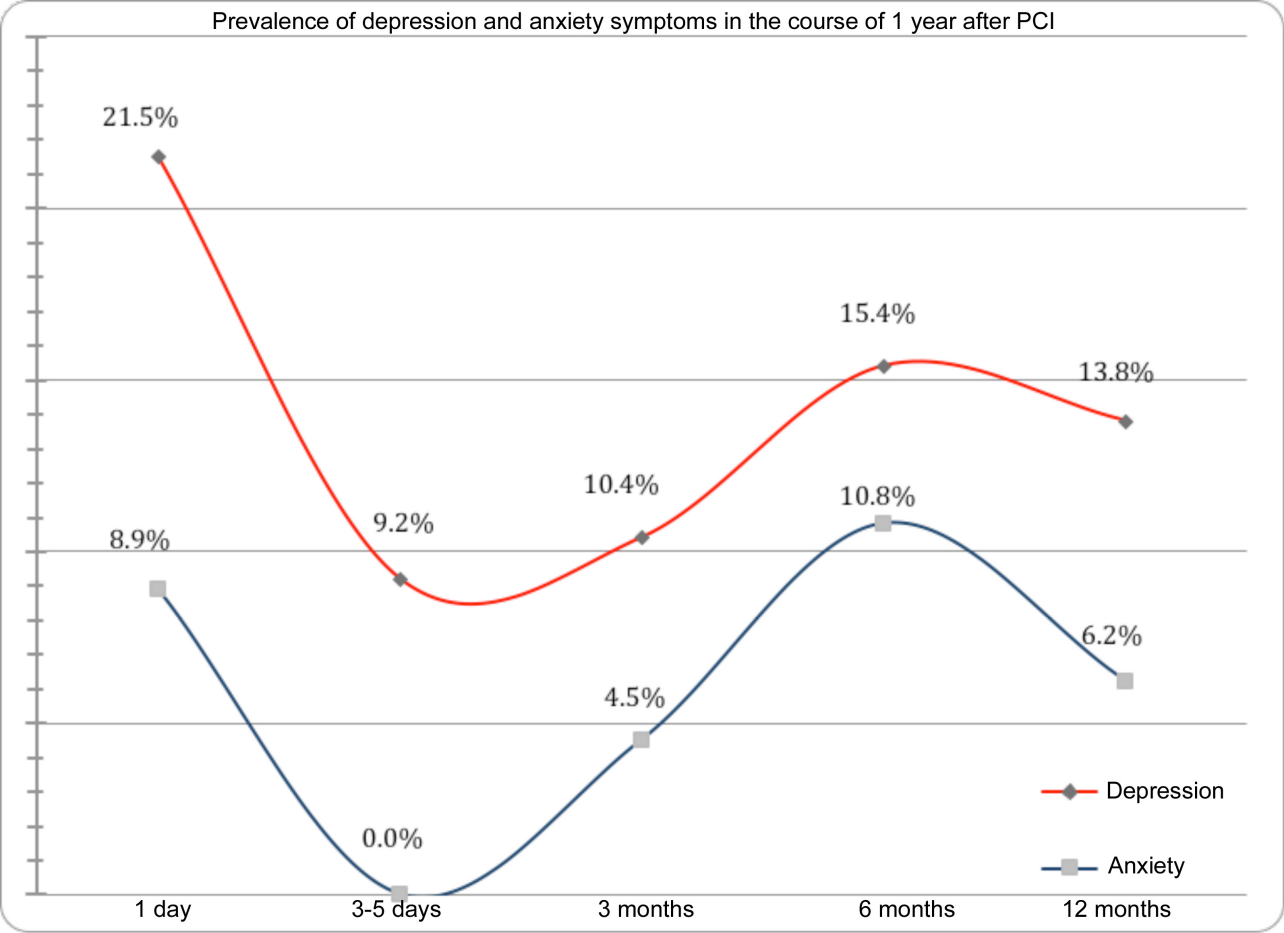
Prevalence of depression and anxiety symptoms in the course of 1 year after PCI.

Both variables statistically significantly correlated in all measurements (r = 0.61 within 24 hours; r = 0.44 before discharge; r = 0.60 in 3 months; r = 0.80 in 6 months; and r = 0.79 in 12 months). (Fig 1)

### Difference between men and women

In determining the differences between men and women it has been found that the groups statistically significantly differed from each other at the time of hospitalization. Women experienced more depression and anxiety then men in the first 24 hours after PCI (p<0.01; p = 0.01), and also more depression before hospital discharge (p<0.01).

### Difference between age groups

In determining a depressive and anxious state of mind, the values of depression symptoms in different age groups statistically significantly (p = 0.02) differed in the first 24 hours after PCI in different age groups. Patients in the oldest age group between 70 and 79 years had the highest score of BDI-II in the first 24 hours after PCI (average = 12.3, SD = 5.0).

### Sleep disturbances and loss of interest in sex

From the BDI questionnaire, we were further interested specifically in two aspects, which we also assessed in terms of their occurrence in patients over 12 months: changes in sleep and loss of interest in sex. On the first day of hospitalization, the values were most extreme. Changes within the ‘minus’ meaning, i.e. “I sleep a little less”, “I sleep less”, or “I wake up one to two hours earlier and cannot fall asleep again” on the first day of hospitalization were stated by 46% of respondents. Before leaving the hospital, a sharp decline in these changes was apparent—to 26% of the respondents. The third, fourth and fifth measurement stabilized at around 30%.

**Fig 2:**
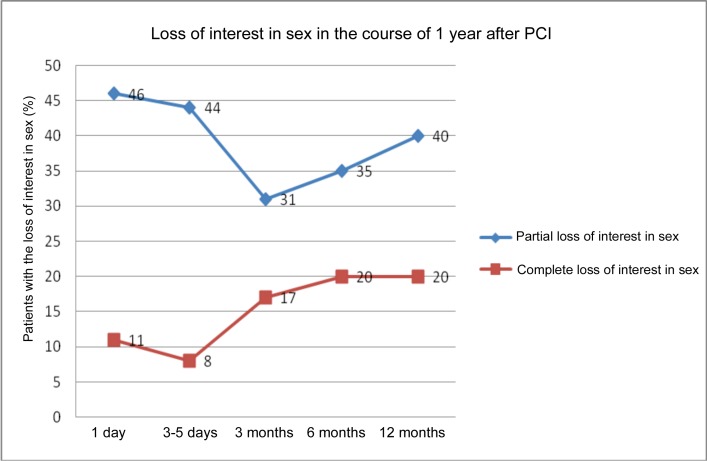
Changes of sleep in the course of 1 year after PCI.

If we compare these sleep disorders with the incidence of depression in our sample, we can see the prevalence of sleep disorders. Changes in the sleep in the sense of “I sleep a little more than usual”, and “I sleep much more than usual” occurred among the respondents during hospitalization in 22%, and before discharge in 17%. Then the values stabilized again around 30%. (Fig 2)

**Fig 3:**
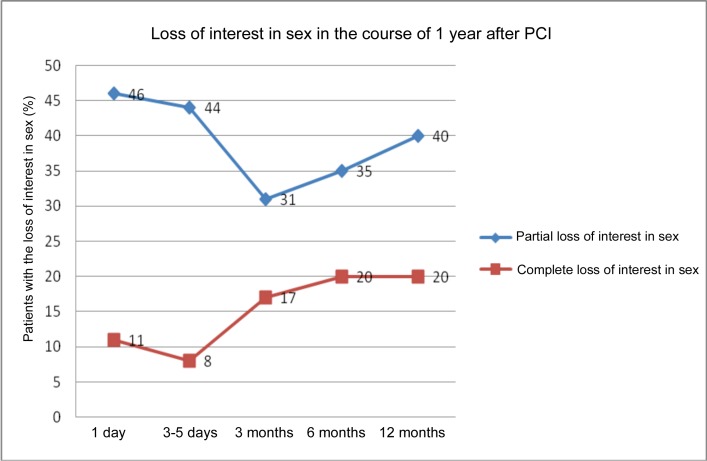
Loss of interest in sex in the course of 1 year after PCI.

Partial loss of interest in sex was reported on the first day by 46%; after the 15% decline, it returned again after 12 months to 40%, with increasing tendency from month 3 to month 12. Complete loss of interest in sex was reported by 11% respondents on the first day, again with increasing tendency. Between month 6 and month 12, the incidence of complete loss of interest in sex stabilized at 20%. (Fig 3)

## Discussion

Unlike previous studies, the prevalence of depression symptoms is lower. In the first 24 hours after PCI, 21.5% of patients with a depressive state of mind were recorded, while after 3–5 days after PCI before discharge from the hospital, the relative number of patients with a depressive state of mind was lower than half (9.2%). This sudden drop in the incidence of depression symptoms in a short time could be explained by mental and/or physical relief after the PCI and disappearance of distressing symptoms of the ischemic heart disease, which could precede AMI. Certain limitation is the unavailability of data prior to the occurrence of MI. Partial information may be provided by first self-rating questionnaires completed within 24 hours after admission, but non-specific distress during acute cardiovascular disease surely burdens it with a certain error. Subsequently, during home treatment, there is an increase in a relative number of patients with depression symptoms—to the values 10.4%, 15.4%, and 13.8% (3, 6, and 12 months after PCI respectively). A similar curve was observed in a separate evaluation of sleep disorders and interest in sex. It can be assumed that it takes some time for the patients to fully realize consequences of severe cardiovascular disease, impaired functional capacity, and in some cases even disability caused by the new disease. This can explain to some extent a gradual increase of the symptoms in home care. In the meta-analytical study[1], 6 similar studies were assessed, ascertaining the prevalence of depression using the BDI (BDI ≥ 10) questionnaire. Averaged relative frequency of the incidence of depression symptoms was 31.1% (CI 29.2%–33.0%; N = 2 273). Our results are significantly lower, after one year one half. This could be partly influenced by using a different (older) version of the BDI questionnaire in these 6 studies.

Prevalence of anxiety is often determined along with depression using the HADS questionnaire (the Hospital Anxiety and Depression Scale), which is divided into two subscales. For example, Furuya et al.[7] found the prevalence of anxiety in 66.7% of men and 56.3% of women, with atypical predominance of the incidence of anxiety in men. Our research group was doing better again in terms of the incidence of anxiety, when we did not use any other method of measurement. Anxiety ranged from 0% to 10.8%. Zero incidence was recorded in the period before leaving the hospital.

Patients reported depression and anxiety symptoms already on the first day after AMI, although the instructions of both self-rating scales required assessment of the condition in the last two weeks. At the same time, a rapid decline was reported immediately after 2–4 days of the completion of the first set of questionnaires. These facts suggest that both BDI-II and SAS evaluated the current condition of depression and anxiety symptoms rather than their average in the last 2 weeks.

Damen et al.[8] mentioned stability of both investigated symptoms at the time after PCI. In this study, the authors focused primarily on the research of changes of depression and anxiety symptoms over time in individual patients separately. They found relative stability of both variables. We came to similar conclusions using Spearman correlation of individual variables at all times. The results at all times were statistically significantly associated with one another.

When evaluating the relationship between depression and anxiety symptoms we came to the conclusion that the values BDI-II and SAS measured at the same time are statistically significantly related. Lane et al.[2] reached the same conclusions. The authors offer two possible explanations of this co-morbidity. These two values are either increased together in response to stress (AMI, hospitalization, PCI, etc.) without relation to any psychopathology, or they represent wrong measurement instruments, which reflect another different psychopathology[2].

By comparing men and women, predominance of depression and anxiety symptoms was explained both in the first 24 hours, and for depression, before the discharge from the hospital. Furuya et al.[7] came to opposite results with their research group. We could explain this disparity in experiencing depression and anxiety symptoms by greater reactivity of women to stressful stimuli.

## Conclusion

Overall, we found a relatively low prevalence of symptoms of depression and anxiety in patients with STEMI treated with primary PCI. A favourable decline in mental stress was observed before the discharge from the hospital, but in a period of one year after PCI, the prevalence of both symptoms gradually increased. This development should be closely monitored in all patients with cardiovascular disease using simple screening methods, such as BDI-II and SAS, which in the clinical practice will allow for rapid evaluation of the risk to the patient and the use of other methods enhancing medical care.
